# The therapeutic characteristics of serial casting on congenital scoliosis: a comparison with non-congenital cases from a single-center experience

**DOI:** 10.1186/s13018-017-0554-7

**Published:** 2017-04-04

**Authors:** Jun Cao, Xue-jun Zhang, Ning Sun, Lin Sun, Dong Guo, Xin-yu Qi, Yun-song Bai, Bao-sheng Sun

**Affiliations:** Orthopedics Department, Beijing Children’s Hospital, Capital Medical University, No. 56 NanLiShi Road, West district, Beijing, China

**Keywords:** Early-onset scoliosis, Congenital scoliosis, Serial casting

## Abstract

**Background:**

The therapeutic efficacy of serial casting on idiopathic scoliosis has been gradually documented. However, literatures on serial casting for congenital scoliosis (CS) remain extremely rare. This paper aimed to compare the treatment outcomes of serial casting between CS and non-CS patients to comprehensively evaluate the therapeutic characteristics of serial casting on CS patients.

**Methods:**

A total of 23 early-onset scoliosis cases were included and divided into congenital scoliosis (CS, *n* = 8) and non-congenital group (non-CS, *n* = 15). Therapeutic outcomes including the major curve Cobb angle, thoracic kyphosis angle, lumbar lodosis angle, and thoracic spine growing rate were compared between groups at precast, after the first cast, and at the latest follow-up, respectively.

**Results:**

All patients received the first cast at the age of 3.25 ± 1.20 years and 5.70 ± 1.18 times of cast corrections. The average casting time was 17.17 ± 3.38 months, and the mean follow-up time was 23.91 ± 12.28 months. Both CS and non-CS groups had significant decrease in Cobb angle after the first cast and at the latest follow-up (all *P* < 0.05). Cobb angle was significantly lower in non-CS group than in CS group at both time points (all *P* < 0.01). The correction rate of Cobb angle was significantly higher in non-CS group than in CS group (around 50 vs. 20%, both *P* < 0.01). The mean thoracic growth rate was significantly lower in CS group than in non-CS group (0.72 ± 0.20 vs. 1.42 ± 0.22 cm/year, *P* < 0.001). At the latest follow-up, there are 2 cases receiving growing rod surgery, 8 cases wearing a brace, and 13 cases continuing serial casting.

**Conclusions:**

Although the therapeutic efficacy of casting on CS patients is not as good as that on non-CS patients, casting is still an efficient treatment option for CS patients to delay the need for initial surgery.

## Background

Early-onset scoliosis (EOS) refers various types of scoliosis with onset before 5 years of age, including neuromuscular, congenital, and syndromic scoliosis [1]. Multiple vertebral deformities-involving congenital scoliosis (CS), neuromuscular scoliosis (NS), various types of syndromic scoliosis and rapidly progressive infantile idiopathic scoliosis (IIS) would quickly progress if not treated. In addition to spinal deformity, progressive scoliosis will also affect the development of thoracic cage and lung, resulting in poor quality of life and long-term prognosis [2–4]. Early control of early-onset scoliosis progress and maintaining the development of thoracic cage and spine are crucial.

Current treatments for EOS include both non-operative and operative techniques. Growing rod surgery is a most widely used operative technique [5]. However, its implant-related complications are frequent [6, 7]. It is suggested that initial implantation of the growing rods should be delayed to reduce the complications of treatment [8]. Non-operative technique is generally considered to be initial treatment for EOS patient if possible, with the purpose to prevent or delay the need for surgical intervention. Casting treatment was first introduced by Risser [9], and Cotrel and Morel modified Risser technique to extension, derotation, flexion (EDF) casting correction [10]. In 2005, Mehta reported the therapeutic effects of EDF casting on 136 IIS cases, demonstrating that patients treated at the early stages of progression (mean age = 19 months) could achieve full correction without further treatment. In patients treated at late stage of progression (mean age = 30 months), EDF casting could only reduce but not reverse the deformity, which also benefits patients by delaying surgery and improving the prognosis [11]. Other studies also reported similar therapeutic outcomes [12–15].

Although the abovementioned reports demonstrated promising therapeutic efficacy of EDF casting, literatures on its therapeutic effect on CS are extremely rare. Non-operative techniques are conventionally considered to have extremely limited effect on CS due to its rigid spinal deformity [16]. Nevertheless, taking advantage of delay of the time of initial surgery, recently serial casting has been adopted in the treatment of CS. Fletcher et al. have reported the therapeutic outcomes of EDF casting correction in 29 EOS patients, including 12 IIS and 17 non-IIS cases. Their results show that 21 patients (72.4%, 21/29) have avoided surgery, and patients need surgery also have an average of 39 months of delay [17]. Very recently, Demirkiran et al. reported their experience of 11 CS patients receiving serial casting. The results show that the major curve Cobb angle and compensatory curve were significantly improved and the mean T1-T12 height also significantly increased at the latest follow-up. Only two patients required growing rod surgery at the latest follow-up, and all patients have an average of 26.3 months of delayed surgery [18]. However, therapeutic efficacy of serial casting on CS was not directly compared with other types of non-CS EOS in the above studies. To comprehensively evaluate the therapeutic characteristics of serial casting on CS, this study aimed to directly compare the treatment outcomes of serial casting between CS (*n* = 8) and relatively flexible non-CS (*n* = 15) cases.

## Methods

### Patients

We retrospectively reviewed the records of all EOS patients receiving serial EDF casting correction at our center from Jan 2010 to Dec 2015. The inclusion criteria were (1) patients continuously received casting treatment for EOS at our center; (2) patients did not received surgical management for EOS prior to casting; (3) the number of cast changes equal to or larger than 3; (4) the casting time larger than 6 months. Exclusion criteria included (1) patients received spinal corrective surgery prior to casting; (2) the number of cast changes smaller than 3; (4) the casting time shorter than 6 months. This study was approved by the institutional review board of our hospital. Written informed consent was obtained from each patient.

### Serial EDF casting correction

Casting was performed under general anesthesia on a casting table using occipital-jaw and pelvic sling to correct scoliosis. A surgeon performed derotation in the apical vertebra area and made a lateral pressure, and two assistants fixed patients’ shoulder and pelvis against the derotation. After hardening, the plaster was trimmed to prevent the impact on physical activity. An abdominal window and a dorsal relief window over the concavity were removed to improve respiratory ventilation and release stress. All casts were of under-the-shoulder modeling.

CS patients with nervous system malformations should be evaluated by a neurosurgeon to assess the need for prior treatment. Spinal flexibility and rotational deformity of CS patients should be assessed by preoperative bending X-rays and spinal three-dimensional CT images before casting. For patients with relatively rigid spine, the correction force should be appropriately reduced to avoid crushing. The derotational force should be appropriately reduced in the patients without severe rotational deformity.

### Data collection

Baseline demographics and clinical characteristics were collected. All patients were followed up regularly, and the cast was changed approximately every 2–4 months according to the age of patients. After completing each session of casting treatment, cast was removed at follow-up and a Cobb standing lateral spine X-ray was taken within 24 h to evaluate the therapeutic effects of casting treatment. Radiographic measurements included scoliosis side, apical vertebra, major curve Cobb angle, thoracic kyphosis angel, lumbar lordosis angel, and T1-T12 height. Correction rate was calculated as follows: (precast angle − post-treatment angle)/precast angle × 100%.

### Final outcomes at the latest follow-up

Final outcomes at the latest follow-up include continued casting correction, control of scoliosis, and surgery. Control of scoliosis was defined as follows: after casting treatment at least 6 times or 18 months, the curvature was reduced to less than 25° and was stabilized within 1-year follow-up. These patients were usually prescribed to continue to wear a brace until skeletal maturity. The indication for surgical management was that scoliosis continued progress after casting treatment at least four times or a year.

### Statistical analysis

For the continuous variables, the mean ± standard deviations (SD) with range (minimum to maximum) were used and were compared with non-parametric statistics including Mann-Whitney *U* and Wilcoxon signed-rank tests. Categorical variables were presented as number and percentage and were compared using chi-square test or Fisher’s exact test. The major curve Cobb angle measures were analyzed by multivariate generalized estimated equation (GEE). The significance level was set at *P* < 0.05. Statistical analyses were performed using SPSS (SPSS Statistics V20, IBM Corporation, Somers, NY).

## Results

### Study subjects

A total of 23 EOS cases (8 CS and 15 IIS patients) receiving casting correction in our center were included. The demographic and clinical characteristics of all patients were summarized in Table 1.

**Table 1:** The demographic and clinical characteristics of each patient

No.	Sex	Age at 1st cast, year	Pathological type	Follow-up, month	Number of casting	Time in cast, month	Top vertebra	Scoliosis side	Cobb angle	Thoracic kyphosis angel	Lumbar lordosis angel	Final outcome
1	F	1.25	CS	25	7	22	T12	Left	68.8	21.3	34.03	Brace
2	F	2.67	CS	19	4	13	T12	Right	88.1	34.9	68.8	Cast
3	M	1.92	CS	13	6	13	T10	Left	55.3	25	54.2	Cast
4	M	3	CS	13	5	13	T9	Left	36.4	31.2	28	Cast
5	M	2.25	CS	47	6	12	T8	Right	66.2	50.9	54.1	Surgery
6	M	5.92	CS	15	4	15	T8	Right	35.7	49.8	45.1	Cast
7	M	4.33	CS	18	5	18	T11	Right	65.8	61.3	66.6	Cast
8	F	3.10	CS	19	6	19	T11	Right	52	36.6	33.5	Cast
9	M	2.83	IIS	14	5	14	T12	Right	53.2	46	41	Cast
10	F	3.5	IIS	41	6	19	T12	Right	36.3	28.9	18.9	Brace
11	F	2.67	IIS	17	6	17	T11	Right	43.8	27.7	21.4	Cast
12	F	3.10	IIS	28	6	22	T12	Left	38.3	31	37.8	Brace
13	F	3	IIS	20	6	19	T7	Right	44.6	19.3	53.5	Brace
14	F	1.33	IIS	16	7	16	T12	Right	35.2	21.2	24.3	Cast
15	F	2.83	IIS	54	8	21	T12	Left	43.2	22.3	39.6	Brace
16	M	3.17	IIS	16	5	16	T11	Right	50.7	39.9	44.2	Cast
17	F	2	IIS	34	7	20	T9	Right	42.5	24.8	33.2	Brace
18	M	3.58	IIS	26	6	20	T12	Left	46.8	24.1	48.2	Brace
19	M	4	IIS	17	5	17	T9	Right	44.3	24.6	15	Cast
20	M	4.83	IIS	14	4	14	T12	Right	38.4	39.6	37.5	Cast
21	F	4.58	IIS	47	8	24	T12	Left	62	35.4	18.9	Brace
22	F	5.5	IIS	23	4	17	T12	Right	68	35.6	34.6	Surgery
23	M	3.5	IIS	14	5	14	T8	Right	87.2	32.6	21	Cast

Sex: *F* female, *M* male. Pathological type: *CS* congenital scoliosis, *IIS* infantile idiopathic scoliosis

To characterize the therapeutic features of serial casting on CS patients, the 23 patients were grouped into CS group and non-CS group. The demographic and clinical characteristics were compared between two groups in Table 2. All patients received their first cast management at the age of 3.25 ± 1.20 years and received 5.70 ± 1.18 times of cast corrections. The mean time in cast was 17.17 ± 3.38 months (12–24, median = 17 months). The mean follow-up time was 23.91 ± 12.28 months (13–54, median = 19 months). As shown in Table 2, there were no significant differences in all the above characteristics and precast Cobb angle, thoracic kyphosis angle between groups (all *P* > 0.05), indicating two groups were comparable.

**Table 2:** The demographic, clinical characteristics, and therapeutic outcomes of patients

Parameters	CS (*n* = 8)	Non-CS (IIS, *n* = 15)	All (*n* = 23)	*P* value^a^
Sex, male	5 (62.50%)	6 (40%)	11 (47.83%)	0.400
Age at 1st cast, year	3.06 ± 1.47 (1.25–5.92)	3.36 ± 1.07 (1.33–5.50)	3.25 ± 1.20 (1.25–5.92)	0.316
Scoliosis side				0.657
Left	3 (37.50%)	4 (26.67%)	7 (30.43%)	
Right	5 (62.50%)	11 (73.33%)	16 (69.57%)	
Follow-up, month	21.13 ± 11.17 (13–47, median 18.5)	25.40 ± 12.96 (14.00–54.00, median 20)	23.91 ± 12.28 (13–54, median 19)	0.382
Number of casting	5.38 ± 1.06 (4–7)	5.87 ± 1.25 (4.00–8.00)	5.70 ± 1.18 (4–8)	0.404
Time in cast, month	15.63 ± 3.62 (12–22, median 14)	18.00 ± 3.05 (14.00–24.00, median 17)	17.17 ± 3.38 (12–24, median 17)	0.086
Cobb angle				
Precast	58.54 ± 17.55 (35.7–88.1)	48.97 ± 13.98 (35.20–87.20)	52.30 ± 15.62 (35.2–88.1)	0.175
After the 1st cast	46.94 ± 15.75 (23.8–65.6)*	26.25 ± 11.95 (13.90–59.60)*	33.45 ± 16.47 (13.9–65.6)*	0.004
The latest follow-up	48.51 ± 14.80 (25.7–64.6)*	27.49 ± 16.86 (4.00–66.20)*	34.80 ± 18.85 (4–66.2)*	0.010
^b^Correction rate—Cobb angle, %				
After the 1st cast	20.52 ± 9.30 (6.83–34.62)	47.53 ± 13.40 (20.17–66.82)	38.14 ± 17.74 (6.83–66.82)	<0.001
The latest follow-up	17.09 ± 9.21 (5.44–28.01)	46.39 ± 21.38 (2.65–90.59)	36.20 ± 22.84 (2.65–90.59)	0.002
Thoracic kyphosis angle				
Precast	38.88 ± 13.88 (21.3–61.3)	30.20 ± 7.85 (19.30–46.00)	33.22 ± 10.88 (19.3–61.3)	0.121
After the 1st cast	34.23 ± 12.80 (17.5–60)	24.79 ± 10.05 (11.00–50.20)*	28.07 ± 11.73 (11–60)	0.071
The latest follow-up	36.21 ± 9.55 (23.7–48.6)	29.71 ± 6.18 (18.60–39.80)^∆^	31.97 ± 7.96 (18.6–48.6)	0.121
Lumbar lordosis angle				
Precast	48.04 ± 15.44 (28–68.8)	32.61 ± 11.96 (15.00–53.50)	37.98 ± 14.95 (15–68.8)	0.033
After the 1st cast	35.08 ± 13.50 (22.03–56.5)	27.74 ± 11.71 (12.70–58.60)	30.29 ± 12.57 (12.7–58.6)*	0.197
The latest follow-up	41.19 ± 10.54 (26.6–58.2)	34.83 ± 8.65 (20.70–49.20)	37.04 ± 9.62 (20.7–58.2)^∆^	0.129
Thoracic spine growth rate, cm/year	0.72 ± 0.20 (0.46–1)	1.42 ± 0.22 (0.98–1.80)	1.18 ± 0.40 (0.46–1.8)	<0.001
Outcome at the latest follow-up				0.168
Cast	7 (75.00%)	7 (46.67%)	12 (52.17%)	
Brace	1 (12.50%)	7 (46.67%)	8 (34.78%)	
Surgery	1 (12.50%)	1 (6.67%)	3 (13.04%)	

Data was presented as mean ± SD (range) or *n* (%)

*P < 0.05 in intragroup comparison to “precast”

^∆^ *P* < 0.05 in intragroup comparison to “after 1st cast”

^a^ *P* values for intergroup comparison

^b^Correction rate was calculated as follows: (precast angle − post-treatment angle)/precast angle × 100%

### Serial casting had better therapeutic effect on non-CS patients than on CS patients

**Fig. 1:**
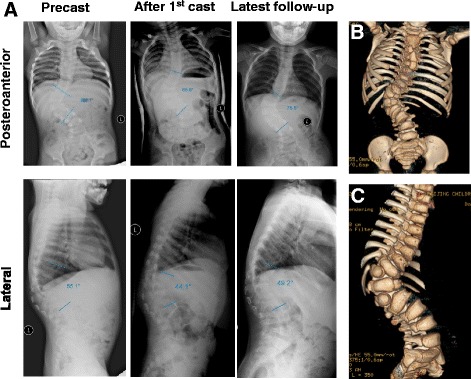
A representative case of CS patient (case no. 2). A 31-month-old female patient visited our clinic for back imbalance. X-ray radiographs revealed congenital scoliosis combined with kyphosis (**a**, *left panels*). Further CT examination revealed two hemivertebrae in thoracolumbar segment (**b**, **c**). The patient weighed 11.3 kg. After the first cast, both the degrees of scoliosis and kyphosis were reduced (**a**, *middle panels*). After four sessions of casting treatment during 13 months, the progress of scoliosis and kyphosis were both controlled (**a**, *right panels*). Patient weight increased to 14.2 kg at the latest follow-up

**Fig. 2:**
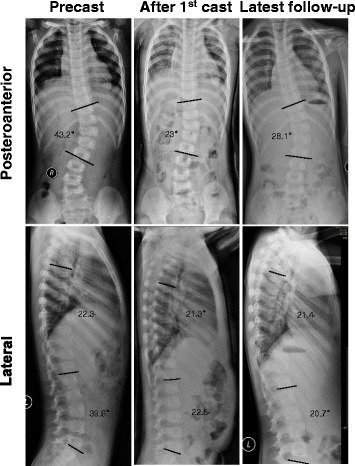
Radiographs of a representative case of non-CS patient (case no. 15). A 13-month-old female patient visited our clinic due to scoliosis. X-ray examination revealed IIS. The patient was unable to receive bracing treatment due to her young age, and the scoliosis continued progress. Therefore, the patient started receiving casting treatment at 2.75 years old. The scoliosis became stable after eight times of casting correction, and then, the patient wore a brace

As showed in Table 2, compared to precast, both CS (Fig. 1) and non-CS groups (Fig. 2) had significant decrease in Cobb angle after the first cast and at the latest follow-up (all *P* < 0.05). A multivariate generalized estimated equation (GEE) model was used to further confirm the therapeutic effect of casting. As shown in Table 3, after adjustment of all other baseline demographic and clinical variables, including age, sex, follow-up time, number of casting, time in cast, and scoliosis side, both Cobb angles after the first cast and at the latest follow-up were significantly higher than that at precast (both *P* < 0.001) in all patients (*n* = 23).

**Table 3:** Multivariate generalized estimated equation (GEE) analysis for Cobb angle

	Cobb angle	
Parameters	*B* (95% CI)	*P*
Time point		
Precast	Ref	
After the 1st cast	−18.85 (−22.12–15.58)	<0.001
At the latest follow-up	−17.50 (−21.62–13.38)	<0.001
Group		
CS	Ref	
Non-CS	−13.57 (−26.38–0.76)	0.038

Multivariate GEE was used to investigate the effects of group and time on Cobb angles with adjustment of the other baseline variables including age, sex, follow-up time, number of casting, time in cast, and scoliosis side

In the intergroup comparison, Cobb angle was significantly lower in non-CS group than in CS group at both time points of after the first cast (*P* = 0.004) and the latest follow-up (*P* = 0.010). Accordingly, the correction rate of Cobb angle was significantly higher in non-CS group than in CS group (both *P* < 0.01). Likewise, multivariate GEE analysis showed that the Cobb angle in non-CS group was significantly lower than that of CS group (*P* = 0.038). These results suggested that serial casting had therapeutic effects of scoliosis on both groups but had a better one in non-CS group.

### Serial casting had no effect on the sagittal spinal curvature

To investigate if serial casting had effect on the sagittal spinal curvature, we measured the angles thoracic kyphosis and lumbar lordosis. Of thoracic kyphosis angle, only non-CS group had a significant decrease after the first cast (*P* = 0.023), but it returned to the level of precast at latest follow-up. No significant difference was found in lumbar lordosis angle at three measured time points in both groups. These results suggested that serial casting had no effect on the sagittal spinal curvature. However, the thoracic spine growth rate of non-CS group was significantly higher than that of CS group (*P* < 0.001).

### Complications and final outcomes

Regarding the complications, five patients (three cases in CS group and two cases in non-CS group) had friction-induced ulcers, which were subsequently cured after medication treatment. One case of vomiting in CS group was resolved by symptomatic treatment. One patient in CS group had continuous elevated airway pressure during casting and cannot resume normal breathing after extubation. After the cast was bivalved to increase the thoracic space, patient’s breathing returned to normal.

As for the outcomes at latest follow-up, in CS group, six (75.00%) patients continued receiving cast correction, and one (12.50%) case had further surgery. The curve was stable and did not progress in one (12.50%) patient which then received bracing treatment. In non-CS group, seven (46.67%) cases continued receiving cast correction, seven (46.67%) cases were of control of scoliosis and prescribed to bracing treatment, and one (6.67%) case had further surgery. There was no significant difference in the final outcome between the two groups (Table 2, *P* = 0.168).

## Discussion

In the current study, we compared the therapeutic outcomes of serial casting between CS and non-CS patients to investigate the therapeutic characteristics of casting on CS. The results showed that non-CS group had significantly lower major curve Cobb angle and a better correction rate of major curve Cobb angle at both time points of after the first cast and the latest follow-up as compared with CS group, suggesting that serial casting had better therapeutic effects on non-CS patients than on CS patients. No significant difference of patient’s final outcome could be observed between the two groups. To our knowledge, this is the first study directly compared the therapeutic effects of casting between CS and non-CS patients.

Comparing therapeutic outcomes between CS and non-CS group, a significant difference could be found. After the first cast, non-CS patients had a good therapeutic outcome. In addition, multivariate GEE analysis also confirmed that the major curve Cobb angle in non-CS group was significantly lower than that of the CS group. The mean correction rate was 20.52 ± 9.30% for CS group, which was far below than that of non-CS patients (47.53 ± 13.40%). Consistently, Demirkiran et al. have reported a similar correction rate of 22% in 11 CS patients receiving casting treatment [18]. It is worth mentioning that even though the difference in precast Cobb angle between groups did not reach statistical significance, the degree of scoliosis was larger in CS group than in non-CS group by about 10° (58.54 ± 17.55 vs. 48.97 ± 13.98, *P* = 0.175). The main reason for this difference is that at our center, CS patients are prescribed serial casting treatment only when they have multiple hemivertebrae, multiple vertebral dysplasia, or even combined with rib fusion, which make the Cobb angle relatively large and rapidly progressive. In addition, the multiple vertebral deformities make the spinal more rigid and more difficult to be corrected by casting. In contrast, non-CS patients have a relative flexible spine, which is more easily to be corrected by the traction and derotational force. Both the larger precast Cobb angle and disease features of CS group contributed to the significant difference in the mean correction rate between CS and non-CS group. Although the therapeutic effect of casting on CS patients was not as good as that of non-CS patients, however, the major curve Cobb angle maintained stable to the latest follow-up, suggesting that serial casting can maintain its therapeutic effects on CS patients and the progress of scoliosis was under control. Regarding thoracic growth rate, all the patients had a mean spinal growth rate of 1.18 ± 0.40 cm/year, which is slightly lower than normal range of 0–5-year children (1.5 cm/year) [19]. The mean thoracic growth rate was significantly lower in CS group than in non-CS group (*P* < 0.001), which may be attributed to the fact that spinal growth rate of CS patients would have been below normal levels due to their vertebral deformity. Under the traction force in casting correction, serial casting correction would help spinal growth according to Hueter-Volkmann law [20]. Therefore, based on our clinical practice, we believe that serial casting is an optimal treatment for CS patients with large scale of vertebra involved, rapid progress, young age (<3 years), and low body weight (below the 5th percentile line [12 kg for 3-year child in China] on the growth chart).

Serial casting has been regarded as an effective treatment to delay the need for first surgery for EOS patients [17, 18]. Fletcher et al. have reported that 8 out of 29 EOS patients receiving casting correction have an average of 39 months of delayed surgery [17]. Likewise, Demirkiran et al. have reported that 11 CS patients receiving serial casting have an average of 26.3 months of delayed surgery [18]. In this study, at the latest follow-up, one CS patient and one non-CS patient received growing rod surgery. Consistent with the aforementioned observations, our results showed that casting correction delayed the time of first surgery by an average of 15 months for these 2 cases. Meanwhile, 6 out of 8 CS cases continued to receive casting treatment. The scoliosis was controlled in 7 non-CS cases, and the angle of scoliosis was still large but stable and not progressive in 1 CS patient. These 8 patients were then prescribed bracing treatment. Although most of these patients may still require surgical intervention eventually, however, serial casting has effectively delayed the need for first surgery. Considering distraction surgery should be conducted every 6 months after the growing rod surgery, casting treatment has prevented more than 70 surgeries for all the 23 patients.

Regarding complications, one case had continuous elevated airway pressure during casting procedure and cannot resume normal breathing after extubation. It has also been previously reported that airway pressure may elevate during cast correction process in some patients [12, 14], which may be more frequently occur in the patients with respiratory disease or abnormality than in normal children [14].

There are still some limitations in the present study. This is a retrospective study with small sample sizes in both groups, and the follow-up times were relatively short. A well-designed prospective study with a large sample size and long-term follow-up is required for further evaluation of the therapeutic effect. In addition, the precast Cobb angle of CS group was larger than that of non-CS group by about 10°, although the difference was not significant. Furthermore, the thoracic height was determined by measuring the distance between T1 to T12 in the spinal radiographs, which may cause error due the change of spinal sagittal angle. Even though three-dimensional CT measurement possesses a higher accuracy, we did not adopt CT due to its disadvantage of multiple radiation exposure. All these limitations should be considered in the following study.

## Conclusions

In summary, our study showed that casting correction can benefit both non-CS and CS patients. Although the therapeutic efficacy of casting on CS patients is not as good as that of non-CS patients, casting is an efficient treatment option for CS patients to delay the need for initial surgery. Our study is helpful to better evaluate the therapeutic values of casting correction on CS patients.

## Abbreviations

CS: Congenital scoliosis
EDF: Extension, derotation, flexion
EOS: Early-onset scoliosis
GEE: Generalized estimated equation
IIS: Infantile idiopathic scoliosis
non-CS: Non-congenital group
NS: Neuromuscular scoliosis
SD: Standard deviations
